# Does trait mindfulness mediate the relationship between borderline personality symptoms and emotion dysregulation?

**DOI:** 10.1186/s40479-023-00225-0

**Published:** 2023-06-08

**Authors:** Alison Roberts, Richard de Visser, Claire Rosten, Helen Startup, Clara Strauss

**Affiliations:** 1 University of Sussex, Brighton and Hove, UK; 2 Sussex Partnership NHS Foundation Trust, Brighton and Hove, UK; 3 Brighton and Sussex Medical School, Brighton and Hove, UK; 4 University of Brighton, Brighton and Hove, UK

**Keywords:** Borderline Personality, Emotionally Unstable Personality, Emotion (Dys)regulation, Mindfulness, Mediation Analysis

## Abstract

**Background:**

Emotion dysregulation is core to many biopsychosocial models of Borderline Personality Disorder (BPD) and is often targeted as part of their associated psychological therapies. Several distinct specialist psychotherapies are thought to be effective for people diagnosed with BPD but it is unclear whether they share common change mechanisms. Some evidence suggests that Mindfulness Based Interventions improve competency in emotion regulation as well as trait mindfulness, which are both plausibly associated with good treatment outcomes. It is unclear whether the association between the severity of BPD symptoms and emotion dysregulation is mediated by trait mindfulness. Would improvement in trait mindfulness mediate an association between lower severity of BPD symptoms and fewer problems of emotion dysregulation?

**Methods:**

One thousand and twelve participants completed online, single time-point, self-report questionnaires.

**Results:**

As predicted, the severity of BPD symptoms was significantly and positively associated with emotion dysregulation with a large effect size (*r* = .77). Trait mindfulness mediated this relationship as the 95% confidence interval for the indirect effect did not cross zero (size of direct effect = .48 and size of indirect effect = .29 [.25, .33].

**Conclusions:**

The relationship between the severity of BPD symptoms and emotion dysregulation was confirmed in this dataset. As hypothesised, this relationship was mediated by trait mindfulness. Process measures of emotion dysregulation and mindfulness should be included in intervention studies for people diagnosed with BPD to understand if improvements in these factors are a universal occurrence with good response to treatment. Other process measures should also be explored to identify other factors involved in the relationship between BPD symptoms and emotion dysregulation.

## Background

As defined in DSM-5 [1], Borderline Personality Disorder (BPD) can be diagnosed when someone experiences a pervasive pattern of instability in their interpersonal relationships, self-image, emotions; and when, in addition, their behaviour demonstrates significant impulsivity. This pattern is deemed to meet diagnostic criteria for a disorder (rather than a trait) when it significantly impairs functioning and is not better explained by other mental health difficulties.

### The role of emotion dysregulation in BPD

A review summarising current understanding of the aetiology and core mechanisms of BPD describes two broad schools of thought [2]. One of these, based on the idea of differences in social cognition, gives rise to intervention via Mentalisation Based Treatment [3]. The second school of thought proposes emotion dysregulation as the factor with a central role and this gives rise to intervention via Dialectical Behaviour Therapy (DBT) [4]. Within cognitive psychology emotion regulation can be broadly defined as the process of influencing the experience and expression of emotions [5].

Dialectical Behavior Therapy (DBT) is arguably the most well evidenced psychological treatment for the symptoms of BPD [6]. It comprises four components of treatment: individual therapy, skills training group therapy, telephone coaching and a team therapy approach. The therapy content has four treatment modules and one of these is focused on emotion regulation. Within this clinical context [4] a more detailed definition of emotion regulation is given as the ability to:

1. inhibit impulsive/inappropriate behaviour related to strong emotions,
2. coordinate action towards an external goal,
3. self-soothe any physiological arousal,
4. refocus attention.

The DBT emotion regulation module structures the teaching of emotion regulation skills into four stages. Namely, understanding and naming emotions, changing unwanted emotions, reducing vulnerability to ‘emotion mind’^1^ and managing extreme emotions.

Linehan’s [4] formulation of the *central role* of emotion dysregulation has some research support. People with a diagnosis of BPD have been found to have emotion regulation impairment compared to people without this diagnosis [7]. A review of 93 studies [8] looking at six of the most commonly used emotion regulation strategies confirmed greater use of ‘maladaptive’ rather than ‘adaptive’ strategies in people diagnosed with BPD compared to both those without a mental health diagnosis and those with different mental health diagnoses. A different review of 55 papers [9] studying choice of emotion regulation strategy (also comparing people with a diagnosis of BPD to people with no, and other, mental health diagnoses) noted strong evidence for the decreased use of cognitive reappraisal and mindfulness by people diagnosed with BPD.

These reviews provide some confirmation of the importance of emotion dysregulation, however there remains a debate as to the *specificity* of this link between BPD symptoms and emotion dysregulation [10]. For example, one review of studies, looking at emotion dysregulation and a range of mental health conditions, found that the vast majority of the 67 studies identified demonstrated a significant post-treatment decrease in emotion dysregulation regardless of the initial type of condition or the type of treatment [11]. This possibly suggests both that emotion dysregulation is not specific to BPD symptoms and that change in emotion dysregulation is not unique to DBT interventions.

Indeed, emotion dysregulation is considered important in the psychological conceptualisation of many mental health conditions. Recent systematic reviews cite it as an evidenced factor in, for example, eating disorders [12], problematic gambling [13], bipolar affective disorder [14], and psychosis [15].

In addition to the debate about specificity, there are differences of opinion as to the extent to which the severity of emotion dysregulation *entirely* explains the severity of BPD symptoms. Despite Linehan [4] describing BPD as a disorder of emotion dysregulation, her model also highlights the importance of other factors. Updated work on her original model [16] elaborates and extends the model. Despite these on-going queries about the specificity and explanatory sufficiency of various aetiological and maintenance factors, emotion dysregulation is one of the defining diagnostic criteria of BPD.

### The relevance of mindfulness to treatments for people diagnosed with BPD

In addition to the emotion regulation module, another of the core modules in DBT teaches mindfulness skills. Indeed, mindfulness has been described as the foundation of the other skills training [4] and has been shown to be associated with decreases in impulsivity [17, 18]. Mindfulness is one of the strategies that may be underused by people with a diagnosis of BPD, either through preference for different strategies or reduced capacity for mindfulness (or both) [9]. It may be that training in this strategy is less available as psychological therapies are often inaccessible for people with a BPD diagnosis [19]. There is evidence that interventions such as DBT can increase the use of mindfulness in people diagnosed with BPD [20, 21].

### What is meant by mindfulness in this context?

One of the challenges in interpreting research evidence in this area is the wide range of activities and interventions described as ‘mindfulness’. Some are described as mindfulness *based* whereas others are more accurately described as mindfulness *informed* [22]. Some research assesses clearly described healthcare interventions delivered in a clinical context to people with specific diagnoses, whilst other research assesses the impact of very brief laboratory-based tasks, often on non-clinical student participants. Thus, it is almost impossible to decipher the necessary and sufficient ingredients of an effective mindfulness intervention from the existing research literature.

Efforts have been made to clarify the essential and optional elements of interventions which constitute a full Mindfulness *Based* Intervention (MBI) to facilitate interpretation and application in this field of research [22]. When focusing on interventions for people with a diagnosis of BPD it is noteworthy that such definitions of MBIs usually exclude interventions such as DBT. DBT can be described as a combination of cognitive, behavioural, and mindfulness based interventions and so would be described as a mindfulness informed intervention.

Within DBT mindfulness is defined as “the act of consciously focusing the mind in the present moment without judgment and without attachment to the moment” (16, p151). The DBT mindfulness module structures the teaching of mindfulness skills as three ‘what’ skills (labelled as observing, describing, participating) and three ‘how’ skills (labelled as non-judgementally, one-mindfully, effectively). The magnitude and quality of the differences between mindfulness-*informed* interventions such as DBT and Acceptance and Commitment Therapy (ACT) and mindfulness-*based* interventions such as Mindfulness Based Cognitive Therapy (MBCT) or Mindfulness Based Stress Reduction (MBSR) are debatable. Eeles and Walker [23] provided an interesting comparison of the way mindfulness is taught in DBT, ACT and MBCT. They argued that mindfulness in ACT focuses on defusion from thoughts, whereas in DBT it focuses on the acceptance of emotional states. They characterised MBCT and MBSR as insight oriented whereas DBT is more of a balance between acceptance and change oriented work. It is important not to overstate the differences between these approaches however, and examination of the session content for all approaches clarifies significant areas of overlap. All three approaches include exercises designed to increase defusion, acceptance and behavioural change.

In summary, the central role and specificity of emotion dysregulation in BPD has been proposed but questioned. The role of mindfulness has been considered but the lack of clarity in the literature about the nature of interventions involving mindfulness muddies the waters. Therefore, exploring the association between the severity of BPD symptoms, problems with emotion dysregulation and lower trait mindfulness may have implications for treatment choice. It may be that both of these aspects require direct intervention, that one may influence the other, or that one may be more important for some people than others.

### The relationship between mindfulness and emotion dysregulation

**Fig. 1:**
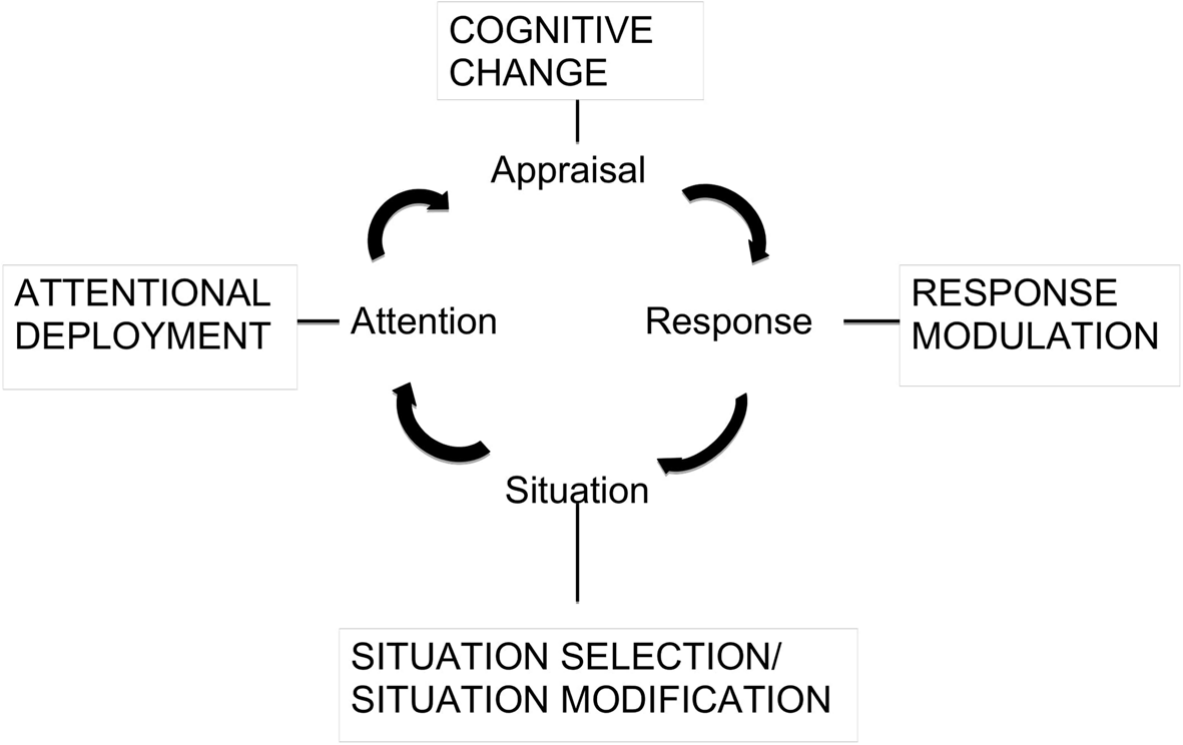
Extended Process Model of Emotion Regulation

Using the extended process model of emotion regulation [5] it has been argued that mindfulness could be valuable in influencing the regulation of emotion at the ‘attention deployment’ stage [24]. It is proposed that this ‘approach’ rather than ‘avoidance’ orientation could disrupt progression on to the stage of unhelpful appraisals and therefore improve emotion regulation. See Fig. 1 [5].

It is of interest that a body of correlational research demonstrates an association between trait mindfulness and BPD. This has been shown for BPD traits in general population participants [25–27] and BPD symptoms in clinical populations [28–30].

Additionally some studies of mindfulness *informed* interventions have found that trait mindfulness [31, 32], or at least certain aspects of it (e.g. acceptance without judgement [33]), can influence DBT treatment outcomes in people diagnosed with BPD.

It has been argued that mindfulness *based* interventions are particularly effective in improving emotion dysregulation in people with affective disorders and increasing self-regulation [24]. This implies that for at least some people, higher trait mindfulness does equate to less emotion dysregulation.

Holzel et al. [34] proposed a number of potential mechanisms of change through which MBIs may be effective, including emotion regulation, but also the processes of attention regulation, body awareness and change in perspective of the self. The authors noted that studies have explored the impact of MBIs on emotion regulation via a range of methodologies including experimental, self-report, peripheral physiological, and neuroimaging data. They concluded that improvements in emotion regulation are associated with mindfulness, and that some emotion regulation strategies may improve after mindfulness practice although it is unclear if this relates to changes in cognitive reappraisal strategies.

However, in regard more tightly defined MBIs there is a paucity of rigorously designed studies. There is little research assessing the implementation of MBIs in people with a diagnosis of BPD, and the results of these are equivocal [35].

Indeed, many questions remain unanswered in this clinically important and interesting area. This includes a full exploration of the extent of the association between BPD symptoms, trait mindfulness, and emotion dysregulation in a treatment-seeking population. Additionally, the extent to which any associations are part of direct, indirect or total effects have largely been unexplored.

### Hypotheses

**Fig. 2:**
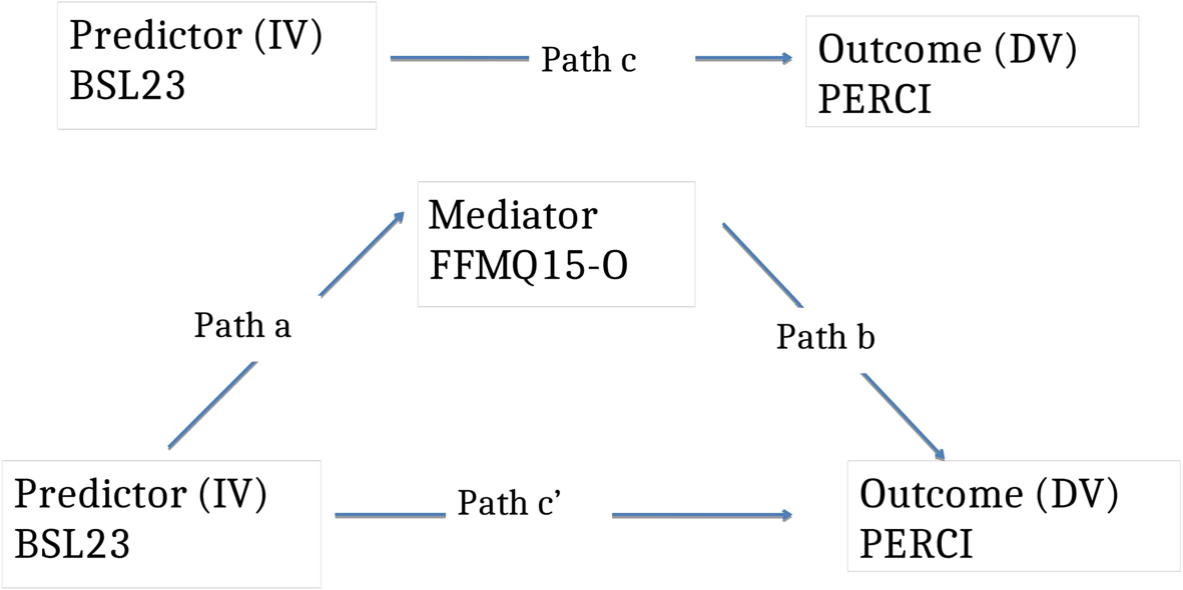
.

The aim of this analysis was to explore the relationship between BPD symptoms and emotion dysregulation and whether any relationship found could be explained by a mediating role of trait mindfulness. The specific hypotheses were:

1. There will be a statistically significant, large, positive correlation between the severity of BPD symptoms and emotion dysregulation as measured by self-report at a single time-point.
2. This correlation will be statistically significant and large in both clinical and non-clinical participants; and in those clinical participants with both low and high BPD symptom severity.
3. Self-reported trait mindfulness will mediate the relationship between the severity of BPD symptoms and emotion dysregulation (see Fig. 2)

As data were collected at a single time point with no temporal sequence, causal inferences cannot be drawn. However there are sound theoretical reasons to suggest that the experiences leading to the development of BPD may inhibit the development of mindfulness skills e.g. a baseline of high emotional sensitivity, an emotionally invalidating environment, the use of other self destructive coping methods. In turn low mindfulness (a lack of ability to notice present moment internal experience non-judgementally) may hinder the development of emotion regulation skills.

## Methods

### Aim, design and setting

The aim of the study was to explore the hypotheses listed above. The data were collected in the United Kingdom’s (UK) National Health Service (NHS) as part of a single time-point cross-sectional survey of online self-report measures. This study is part of a PhD submission exploring emotion regulation, mindfulness and BPD traits.

### Participants

Clinical participants were recruited from people accessing primary care mental health services (Improving Access to Psychological Therapy – IAPT) as this group has been found to include those meeting diagnostic criteria for BPD [36]. Current BPD symptom severity was used rather than BPD diagnostic status as only a subgroup of people who *would* meet criteria for BPD are actually given and made aware of this as an official diagnosis. Participants were not asked whether they had any psychiatric diagnoses. A non-treatment-seeking comparator group of participants was recruited from NHS staff. Recruitment from this population was proposed as a more representative proxy for the general population in terms of broad demographics [37] compared with a convenience student sample, whilst remaining a relatively accessible group of research participants.

Inclusion criteria for the non-treatment-seeking group: any NHS staff, not limited to clinicians and not limited to people below clinical cut-offs on mental health measures (described in the materials section below) as the aim was to provide a proxy for the general population.

Exclusion criteria for the non-treatment-seeking group: if staff were accessing primary care mental health treatment they were moved into the clinical group, if staff were accessing other kinds of mental health treatment they were removed from the dataset.

Inclusion criteria for the clinical group: people awaiting or receiving treatment in NHS IAPT services in England. They were recruited after being accepted in to the service in order to confirm that they were eligible for primary mental health care. The clinical participants were then divided into two groups of high/low level of BPD symptoms dependent on their BSL-23 score (detailed in the materials section below).

The final dataset comprised 1,012 participants who were 77% female, 21.5% male and 1.5% another term or who preferred not to say. Age and ethnicity distributions are presented in Table 1. Fuller sociodemographic data are available in the supplementary information (10.25377/sussex.22561135).

**Table 1:** Age and ethnicity groupings of participants

Characteristic	Non Treatment Seeking *N* = 409		Treatment Seeking Low BPD *N* = 333		Treatment Seeking High BPD *N* = 219		**Full sample** ***N* = 1012**	
	*n*	%	*n*	%	*n*	%	***n***	**%**
Age Group								
18–29	90	22.0	75	22.5	91	41.6	**267**	**26.4**
30–44	133	32.5	117	35.1	71	32.4	**337**	**33.3**
45–59	154	37.7	103	30.9	43	19.6	**314**	**31.0**
60 +	31	7.6	38	11.4	14	6.4	**92**	**9.1**
prefer not to say	1	0.2	0	0	0	0	**2**	**0.2**
Ethnicity								
Asian/ Asian British	16	3.9	13	3.9	6	2.7	**38**	**3.8**
Black/ African/ Caribbean/ Black British	14	3.4	8	2.4	7	3.2	**31**	**3.1**
Chinese/ Chinese British	3	0.7	3	0.9	1	0.5	**7**	**0.7**
Mixed Ethnicity	9	2.2	8	2.4	3	1.4	**20**	**2.0**
Other	4	1.0	7	2.1	3	1.4	**14**	**1.4**
White (British)	332	81.2	265	79.6	174	79.5	**810**	**80.0**
White Other	30	7.3	27	8.1	24	11.0	**86**	**8.5**
prefer not to say	1	0.2	2	0.6	1	0.5	**6**	**0.6**

Exploration of demographic data identified a significant association between gender and allocated group (Pearson Chi square [2] 18.42, *p* < 0.001). This demographic was explored due to the higher ratio of women to men in BPD clinical services that is not replicated in general population studies [38]. Standardised residuals indicated that this difference was not due to different gender proportions in the low and high BPD clinical groups but was a result of fewer men than would be expected in the non-treatment seeking NHS staff group. The Cramer’s V statistic of 0.139 suggests only a small association between gender and allocated group. Therefore, women were not over-represented in the high BPD clinical group in this dataset.

There was also a significant association between age and allocated group (Pearson Chi square [6] 43.76, *p* < 0.001). In the non-treatment seeking NHS staff group significantly more participants were between 45 and 59 than would be expected. The distribution of age in the clinical low BPD group was as expected, but in the clinical high BPD group significantly more participants than expected were between 18 and 29 and significantly fewer were between 45 and 59. This dataset is consistent with the literature [6] suggesting a peak of BPD symptoms in later adolescence/early adulthood.

### Materials

The 23-item self-report *Borderline Symptom List (BSL-23)* was used to measure the level of BPD symptoms and is a shortened version of the BSL-95 [39]. An empirically-based severity classification [40] is based on the total score and suggests a moderate severity level of 1.5 as a cut-off for people with BPD from a mixed clinical control. A mild severity level of 0.64 discriminates people with BPD from non-clinical controls. Bohus et al. [39] reported internal consistency as high (0.935 – 0.969), with the ability to discriminate people diagnosed with BPD from people diagnosed with Axis-I disorders at a mean effect size of 1.13. In the current study Cronbach’s α was 0.960 in the total sample.

The recently developed *Perth Emotion Regulation Competency Inventory (PERCI),* with 32 items rated on a 7-point Likert agreement scale, was used to measure self-reported emotion dysregulation. Higher emotion dysregulation (i.e., poorer emotion regulation competency) is indicated by a higher score [41]. A number of subscales and composite scores can be calculated including the regulation of negative emotions in comparison to the regulation of positive emotions. The total score (General Emotion Regulation) is used in this analysis. This hierarchical structure has been confirmed by CFA analysis of the measure in general population and clinical populations. (Roberts et al. in prep). Preece et al. [42] reported excellent reliability with Cronbach’s α of 0.94 from a general population sample. In the current study Cronbach’s α was 0.956.

A shortened version *(FFMQ-15) *[43] of the original 39-item *Five Facet Mindfulness Questionnaire* [44] was used to measure trait mindfulness comprising 15 self-report items covering five different facets of mindfulness – observing, describing, acting with awareness, non-judging of inner experience, and non-reactivity. Recent analysis [45] has proposed that using a total score without the ‘observing’ subscale is more psychometrically robust so this scoring was used (called FFMQ-15-O to avoid confusion). The FFMQ-15 has been proposed [46] as the most suitable measure of trait mindfulness in people diagnosed with personality disorders. The reliability of each of the subscales has been reported as good (between 0.75 and 0.91) and the total score is reported as sensitive to change. In the current study FFMQ-15-O had a Cronbach’s α of 0.856.

### Data collection process

Ethics approval was gained (see declarations section below) and staff participants were then recruited via NHS internal communication routes. Clinical participants were recruited via emails to people on treatment waiting lists in eleven NHS Trusts around England. Remote recruitment was conducted as services were being delivered remotely during Covid-19 precautions. Recruitment information provided a digital link to a Qualtrics site hosting the self-report measures where participants were provided with sufficient information to ensure voluntary informed consent. Data were downloaded into IBM SPSS (version 27) once fully submitted.

### Analysis plan

Data were cleaned and checked; full details of data management and the approach taken to the very small amount of missing data are given in the supplementary information (10.25377/sussex.22561135).

Hypotheses 1 and 2 were tested using a Pearson’s r correlation coefficient in IBM SPSS (version 27). Hypothesis 3 was tested with mediation analysis, the aim of which was to test whether there is an indirect relationship between BPD symptoms (predictor variable) and emotion dysregulation (outcome variable) through trait mindfulness. This was tested using bias corrected bootstrapped mediation analysis with 5,000 resamples using model 4 of Hayes’ Process command [47], also in IBM SPSS (version 27).

The choice of analysis was informed by a review of psychology research comparing methods of mediation analyses [48], which also included calculations of necessary sample sizes across some of the different methods. This review concluded that bias-corrected bootstrap had the highest power of a number of different analytic strategies. A priori power calculations for mediation analysis were not completed, as the primary use of the data collection was a psychometric and confirmatory factor analysis of the PERCI. A post hoc check of statistical power was completed for this study to confirm the data had sufficient sensitivity in a null hypothesis test to detect an effect (if an effect were present). The necessary sample size for power of 0.8 using bias corrected bootstrapping methods (where the effect size of path a is large and the effect size of path b is medium) was N = 54 and so the sample size of 1,012 for this dataset is clearly sufficient. The minimum effect size that could be detected with the current sample size and a power of 0.95 would be 0.113. The choice of bias corrected bootstrapping also allows for some violation in the assumptions of the data required for general linear models [47].

## Results

### Data exploration

Table 2 provides the mean and standard deviation for all variables for the overall dataset and the participants divided by allocated group. These descriptive data were in the direction expected in that mindfulness was lower and emotion regulation higher in the clinical group than the non-treatment seeking group and higher in the high BPD clinical group than in the low BPD clinical group.

**Table 2:** Means and standard deviations for all variables for the total dataset and by allocated group

Variable	Non Treatment Seeking *N* = 409		Clinical Low BPD *N* = 333		Clinical High BPD *N* = 219		**Full sample** ***N* = 1012**	
	*mean*	sd	*Mean*	sd	*mean*	sd	*mean*	sd
BSL-23	0.494	0.582	0.789	0.384	2.321	0.557	**1.019**	**0.885**
PERCI	74.071	27.639	97.586	27.744	132.128	25.132	**95.600**	**34.487**
FFMQ-15—O	42.548	7.622	36.598	6.901	29.110	6.268	**37.382**	**8.804**

With regard to the data assumptions required for all planned analyses, it is noted that the data were interval data, the relationship between the predictor and outcome variable was linear, and each participant had a data point for each of the variables. As would be expected from a sample including a non-treatment seeking group, the measure of BPD symptom severity was not normally distributed. The measures of trait mindfulness and emotion regulation capacity were more normally distributed. Histograms and boxplots of total scores on all measures are included in the supplementary information (10.25377/sussex.22561135).

The large sample size means that significance tests of skew, kurtosis, homogeneity of variance are inappropriate [49]. As there was some skew present in the data, a non-parametric Spearman’s rho correlation analysis was conducted as a sensitivity check as this assumes only ordinal data and monotonicity. This supported the main analysis (reported in detail below) showing a significant correlation with a large effect size (r_s_ = 0.783 *p* < 0.001 95% BCa CI [0.757, 0.806]). As noted in the analysis plan bias corrected bootstrapping also allows for some violation in the data assumptions. The independence of errors (i.e., whether the residuals are independent of one another) was explored by plotting standardised predicted values against standardised residuals. VIF values between 1 and 5 indicated that multicollinearity was not high enough to be problematic.

### Correlations

Hypothesis 1 – *there will be a statistically significant and large positive correlation between the severity of BPD symptoms and emotion dysregulation.*

**Fig. 3:**
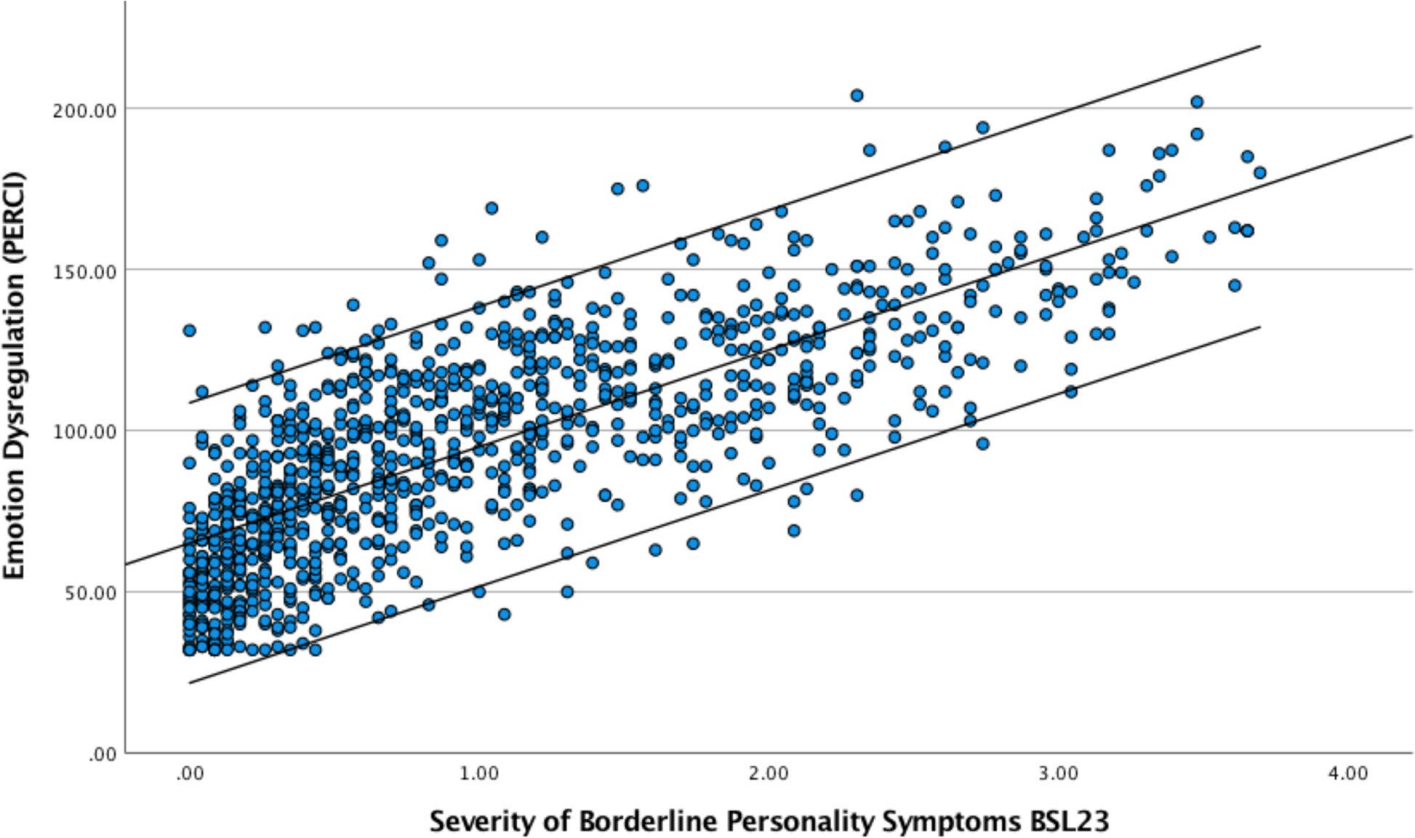
.

Initial exploration of the relationship between the severity of BPD symptoms and emotion dysregulation in the whole sample confirmed this hypothesis. This is demonstrated visually in Fig. 3. To quantify the extent of the correlation between these two variables the Pearson correlation coefficient for the whole sample was calculated and this confirmed a significant positive association with a large effect size, *r* = 0.77 *p* < 0.001 95% BCa CI [0.74, 0.79]. The coefficient of determination (r^2^) indicated that 59% of the variance in BPD symptoms was shared with emotion dysregulation.

Hypothesis 2 – *this correlation will be significant and large in all allocated groups.*

**Fig. 4:**
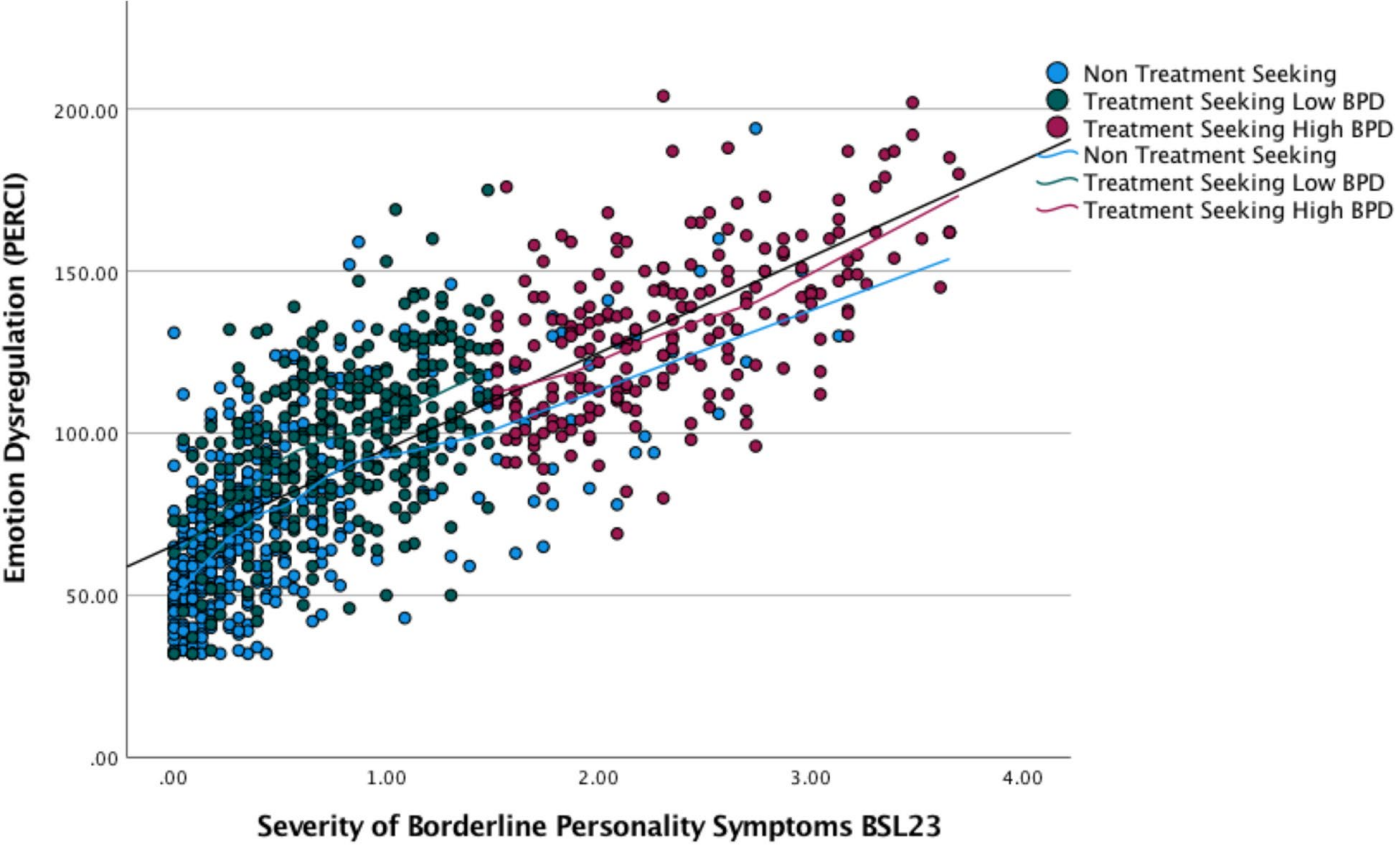
.

The second hypothesis was confirmed by further exploration of the relationship between the severity of BPD symptoms and emotion dysregulation when the participants were divided in to three groups. The correlation between the variables is demonstrated visually in Fig. 4.

Table 3 provides details of the Pearson correlation coefficient for the three separate groups, all of which had a significance level of p < 0.001. As hypothesised there was a significant positive correlation between the severity of BPD symptoms and emotion dysregulation with a large effect size in all three groups. The r value for this relationship differed between the three groups.

**Table 3:** Pearson correlation coefficient, significance level, bias corrected boot strapped 95% confidence intervals and coefficient of determination for the three groups

Group	r	p	Bias corrected boot strapped 95% confidence intervals	r^2^ % of shared variability
Non-treatment-seeking	.635	.001	.572, .689	.403
Treatment-seeking High BPD	.520	.001	.437, .594	.271
Treatment-seeking Low BPD	.576	.001	.479, .657	.331

The extent of these differences can be quantified by transforming the r values to z sores and a statistical website which calculates differences between correlations [50] was used to confirm that (with a two tailed hypothesis) p values of Fisher’s Test indicated that the correlation in the low BPD group (0.520) was not significantly different from the correlation in the high BPD group (0.576) and that the correlation in the high BPD group (0.576) was not significantly different from the non-treatment-seeking group (0.635). However, the correlation in the low BPD group (0.520) was significantly different from the correlation in the non-treatment-seeking group (0.635). Zou’s confidence interval analysis confirmed this pattern of results. This suggests that in this dataset the relationship between BPD symptoms and emotion dysregulation was largest in the non-treatment-seeking group but that this difference was only significantly different when compared to the low BPD group. Notably, all these values indicate a large effect size so do not affect the confirmation of the hypothesis.

### Mediation analysis

Hypothesis 3 – *self-reported trait mindfulness will mediate the relationship between the severity of BPD symptoms and emotion dysregulation.*

**Fig. 5:**
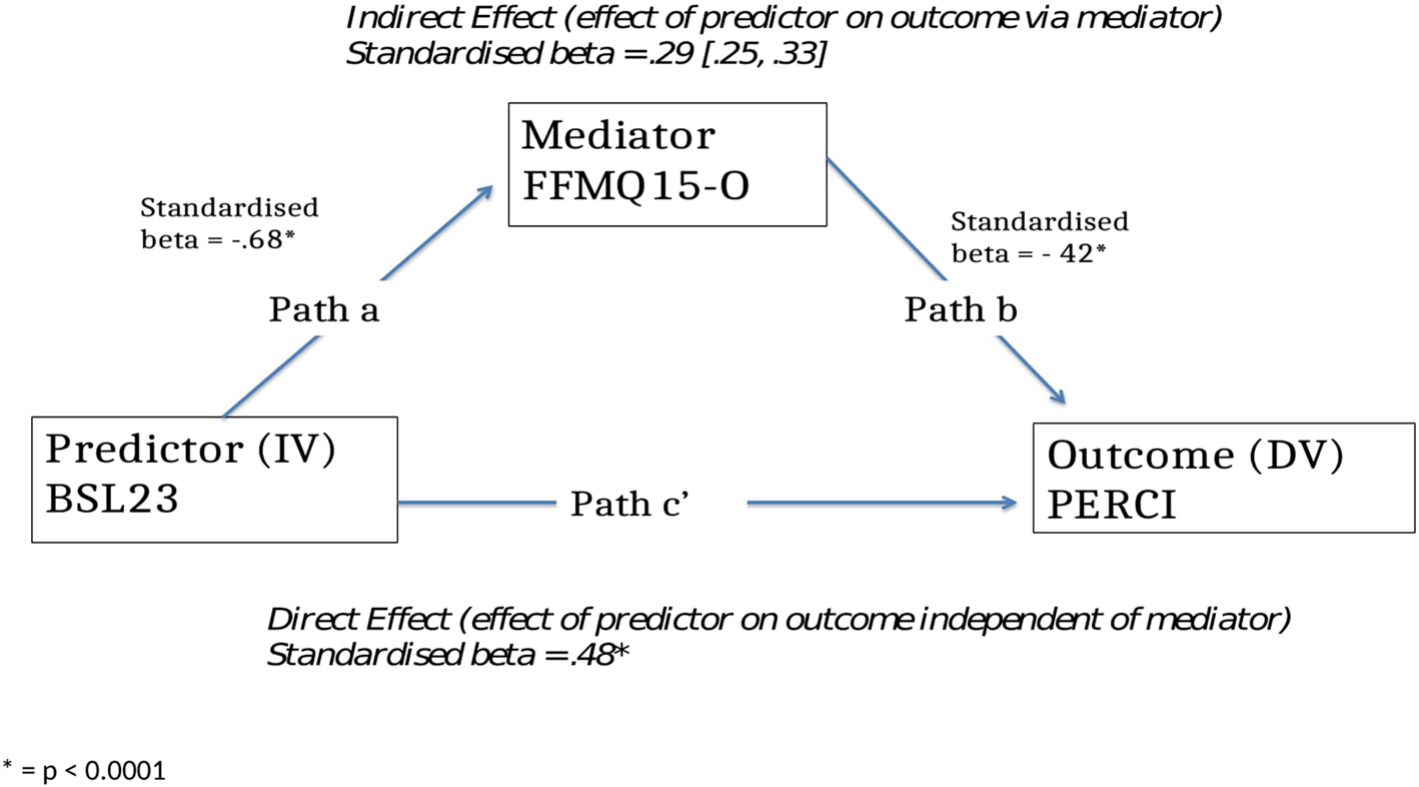
.

**Fig. 6:**
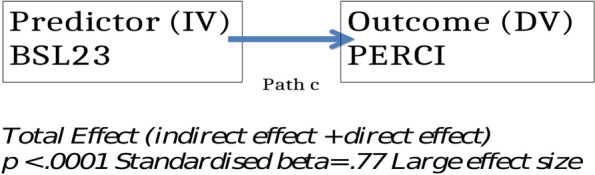
.

Mediation analysis built on these simple correlations by the addition of trait mindfulness as a mediator variable to test hypothesis 3. Figures 5 and 6 provide visual representations and Table 4 summarises the statistics for the different paths.

**Table 4:** Statistics for the different paths in the mediation analysis

Path	b BCa CI	Standardised beta coefficient	significance
a—the effect of the predictor on the mediator	.679 [-.724, -.635]	- .68	*p* < 0.0001
b—the effect of the mediator on the outcome *whilst controlling for the predictor*	-1.66 [-1.85, -1.48]	- .42	*p* < 0.0001
c' the effect of the predictor on the outcome *independently of the mediator*	18.62 [16.78, 20.47]	- .48	*p* < 0.0001
Indirect effect	11.29 [9.81, 12.89]	.29 [.25, .33]	
c—the effect of the predictor on the outcome	29.92 [28.38, 31.46]		*p* < 0.001

Analysis of path a (the effect of the predictor on the mediator) indicated that the severity of BPD symptoms predicted trait mindfulness. This was in the predicted direction of higher BPD symptoms associated with lower trait mindfulness (b =—0.679 BCa CI [-0.724, -0.635], *p *< 0.0001). The standardised beta coefficient of -0.68 indicates a large effect size.

Analysis of path b (the effect of the mediator on the outcome *whilst controlling for the predictor*) indicated that trait mindfulness predicted emotion dysregulation whilst controlling for severity of BPD symptoms. This was in the predicted direction of lower trait mindfulness associated with higher emotion dysregulation regardless of severity of BPD symptoms (b = -1.66 [-1.85, -1.48], *p* < 0.0001). The standardised beta coefficient of -0.42 indicates a medium to large effect size.

Analysis of the indirect effect (the effect of the predictor on the outcome through the mediator) revealed that the 95% confidence intervals did not include zero, i.e., the severity of BPD symptoms significantly predicted emotion dysregulation through levels of trait mindfulness (b = 11.29 BCa [9.81, 12.89]). Poor trait mindfulness explained, in part, the relationship between BPD symptoms and emotion dysregulation. The standardised beta coefficient (B = 0.29 [0.25, 0.33]) indicated a medium effect size.

The above analysis suggested that trait mindfulness mediates the effect of BPD symptoms on emotion dysregulation in support of hypothesis 3. This mediation may only be partial as the c’ path remains significant when controlling for the mediator. However, this may be an effect of a large sample size [47].

## Discussion

As hypothesised the data showed a statistically significant, positive correlation between the severity of BPD symptoms and emotion dysregulation with a large effect size. The association between these variables remained large for participants who were non-treatment seeking or treatment seeking. It also remained whether they were below or above the clinical cut-off for severity of BPD symptoms.

Subsequent analysis indicated that trait mindfulness mediated the relationship between BPD symptoms and emotion dysregulation. These findings are concordant with the correlational research described in the introduction demonstrating an association between high levels of BPD symptoms (in clinical and non-clinical populations) and both lower trait mindfulness and higher emotion dysregulation. These findings have confirmed these associations using a comprehensive theory-based measure of emotion dysregulation and a measure of trait mindfulness, which has been recommended for use in people with a diagnosis of BPD.

The study also builds on work looking at treatment-relevant factors which might mediate the relationship between BPD symptoms and emotion dysregulation, such as psychological needs frustration [51], depressive rumination [52], mentalising [53] and experiential avoidance [54].

Interestingly a recent randomised control trial [55] which found the mindfulness module of DBT had a statistically greater effect in reducing BPD symptoms compared to the interpersonal effectiveness module of DBT included an analysis exploring if decentering or emotion dysregulation mediated this change. The path analysis suggests that the reduction in BPD symptoms in participants who had attended the DBT mindfulness module was mediated by decentering skill but not by emotion dysregulation. However a serial path analysis including both of these mediators suggested that participation in the DBT mindfulness module reduced BPD symptoms by increasing decentering skill, which subsequently decreased emotion regulation. Measures for outcome and mediation were only collected pre and post intervention however, which is not the ideal time sequence.

### Limitations of study

As this study used data collected at a single time point the analysis is correlational. This limits the conclusions that can be drawn about causality as the mediator was measured contemporaneously rather than prior to the outcome [56]. Causation cannot be determined from cross-sectional data.

All of the data were collected via self-report measures. The lack of data from others (i.e., clinician-rated severity of BPD symptoms) or triangulation with other types of data, for example physiological measures of emotion dysregulation or experimental tasks indicative of capacity for mindfulness, is to some extent a limitation. The BSL23 has been established as a proxy for assessing BPD diagnostic criteria, as outlined in the measures section, but it was designed to measure change in some BPD symptoms. The Clinical High BSL group may include participants with symptoms that overlap with BPD (such as broader emotion distress) without fully meeting BPD diagnostic criteria. However a higher, more conservative cut-off was used to try to minimise this.

As with most research in this area, the study is limited by a poor understanding of how ethnicity and culture interact with conceptualisation and measurement of the key variables. Similarly, the influence of gender on the key variables is also poorly understood. However, the confirmation of Hypotheses 1 and 2 remained valid when the analysis was re-run with male and female participants compared (full details in supplementary information Sussex Figshare link).

### Strengths of study

Despite the reservations above, the data benefit from being collected from a large number of participants who were more representative of the UK population than many student sample studies and the relative success of this with regard to ethnicity and age range can be seen in the supplementary information (10.25377/sussex.22561135). Equally, the range of participants is beneficial in terms of being both treatment and non-treatment seeking, as well as low on the BSL23 (highly unlikely to meet diagnostic criteria for BPD) and high on the BSL23 (highly likely to meet diagnostic criteria for BPD).

### Implications for future research

As per the limitations above, future research could build on this study by triangulation of the self-report data with other ways of measuring the latent concepts. Similarly, a study design measuring the same variables before, during and after an intervention designed to increase trait mindfulness in people with a diagnosis of BPD would inform conclusions about causality, as has already been done in people with other mental health difficulties [24]. Longitudinal data gathered from adolescents with increased risk factors for development of borderline features would also be informative.

Emotion dysregulation and trait mindfulness are both concepts that are transdiagnostic and cross the boundary of ‘clinical’ and ‘non-clinical’ populations. Further research could usefully involve participants with different mental health problems where emotion dysregulation is proposed to be a significant factor e.g., (as described in the introduction) eating disorders, psychosis, bipolar affective disorders, or gambling. Such research could help to clarity whether MBIs reduce emotion dysregulation more broadly, or whether the effects vary according to the types of mental health difficulties people are experiencing.

Research in different countries or with people from different ethnicities and cultures would help to identify the possible impact of these factors.

Given the known heterogeneity and high levels of comorbidity in people with a diagnosis of BPD, there is a danger in only reporting average scores in groups of participants. The sole use of averages may hide different patterns of response that are present within different sub groups of participants. For example, the relationship between the severity of BPD symptoms and trait mindfulness/emotion dysregulation may be different for people with particularly poor mentalisation, high impulsivity or presence of re-experiencing or dissociation triggered by traumatic memories. All of these factors are thought to be important to the aetiology and maintenance of BPD but their universality in this clinical group is debated [16].

### Clinical implications

This last point leads on to consideration of the clinical implications. One of the main reasons for exploring how BPD symptoms are associated with mindfulness and emotion dysregulation is to increase understanding of which psychological therapies might be most effective for whom. Among people diagnosed with BPD this question is complicated by the heterogeneity of their presentations.

Mindfulness *based* and mindfulness *informed* interventions may aim to decrease emotion dysregulation by increasing knowledge and competency of mindfulness strategies. The analysis from this dataset indicates that this may be the case as the relationship between BPD symptoms and emotion dysregulation is mediated by trait mindfulness. Therefore people with a diagnosis of BPD may benefit from mindfulness training as one way of improving their emotion dysregulation and decreasing their BPD symptoms. Some existing supportive research is summarised in two review papers [23, 35] although it is important to note that several of the studies included in these reviews are multiple secondary analyses of the same participants. Despite this limitation these data do suggest that some facets of mindfulness such as accepting without judgement/non-judging and non-reactivity as well as decentering, [55] are beneficial to emotion dysregulation and BPD symptoms.

As with any extension of therapy outside of the existing evidence base close attention should be paid to acceptability, effectiveness and safety as there is limited information about mindfulness based interventions for people diagnosed with BPD [35].

The confirmation of the association between BPD severity, emotion dysregulation and trait mindfulness does indicate that regular clinical use of measures of trait mindfulness and emotion dysregulation may be useful. They could be informative both in suitability screening, treatment process, and treatment outcome measures for clinicians, as well as for researchers developing MBIs for people with a diagnosis of BPD.

Given the strength of the relationship between BPD symptoms and emotion dysregulation, and the complexity and stigma around BPD diagnoses [57], one option for increasing accessibility and acceptability of psychological therapies would be to structure care pathways around the transdiagnostic process of emotion dysregulation rather than the more commonly used structure of psychiatric diagnosis, such as with NICE guidelines in the UK [38].

## Conclusions

In summary, this paper confirms in a UK population a statistically significant, large association between the severity of BPD symptoms and emotion dysregulation irrespective of mental health treatment-seeking status. This relationship is mediated by levels of trait mindfulness. Although limited by the correlational and self-report design the results suggest the measurement of trait mindfulness and emotion dysregulation can inform both clinical practice and intervention development. 

## Data Availability

The datasets analysed during the current study are available in the University of Sussex Figshare [LINK].

## Abbreviations

ACT: Acceptance and Commitment Therapy
BPD: Borderline Personality Disorder
CBT: Cognitive Behavioural Therapy
DBT: Dialectical Behavior Therapy
DSM5: Diagnostic and Statistical Manual
ICD11: International Classification of Disease
MBCT: Mindfulness Based Cognitive Therapy
MBI: Mindfulness Based Intervention
MBSR: Mindfulness Based Stress Reduction
MBT: Mentalisation Based Therapy
NHS: National Health Service
NICE: National Institute for Health and Care Excellence

## Footnotes

1 ‘emotion mind’ is defined in DBT as when someone’s thinking and behaviour are controlled by their current emotional state.

## References

[1] American Psychiatric Association (2013). Diagnostic and Statistical Manual of Mental Disorders (DSM-5®).

[2] Winsper C (2018). The aetiology of borderline personality disorder (BPD): contemporary theories and putative mechanisms. Curr Opin Psychol.

[3] Bateman AW, Fonagy P, Campbell C. Mentalization‑based treatment. In: Livesley WJ [Ed, Larstone R [Ed, editors. Handbook of personality disorders: Theory, research, and treatment (2nd ed). New York: The Guilford Press; 2018. p. 541–54, Chapter xv, 712 Pages.

[4] Linehan MM (2015). DBT® skills training manual.

[5] Gross JJ (2015). The extended process model of emotion regulation: elaborations, applications, and future directions. Psychol Inq.

[6] Storebo OJ, Stoffers-Winterling JM, Vollm BA, Kongerslev MT, Mattivi JT, Jorgensen MS (2020). Psychological therapies for people with borderline personality disorder. Cochrane Database Syst Rev.

[7] Gratz KL, Dixon LJ, Kiel EJ, Tull MT (2018). Emotion regulation: Theoretical models, associated outcomes and recent advances. The SAGE handbook of personality and individual differences: Applications of personality and individual differences. Sage Reference.

[8] Daros AR, Williams GE (2019). A meta-analysis and systematic review of emotion-regulation strategies in borderline personality disorder. Harv Rev Psychiatry.

[9] Sorgi-Wilson KM, McCloskey MS. Emotion regulation strategies among individuals with borderline personality disorder relative to other groups: a review. Clin Psychol Psychother. 10.1002/cpp.2738. 10.1002/cpp.2738 35366040

[10] Fitzpatrick S, Varma S, Kuo JR (2020). Is borderline personality disorder really an emotion dysregulation disorder and if so, how? A comprehensive experimental paradigm. Psychol Med.

[11] Sloan E, Hall K, Moulding R, Bryce S, Mildred H, Staiger PK (2017). Emotion regulation as a transdiagnostic treatment construct across anxiety, depression, substance, eating and borderline personality disorders: a systematic review. Clin Psychol Rev.

[12] Leppanen J, Brown D, McLinden H, Williams S, Tchanturia K (2022). The role of emotion regulation in eating disorders: a network meta-analysis approach. Front Psychiatry.

[13] Velotti P, Rogier G, BeomonteZobel S, Billieux J (2021). Association between gambling disorder and emotion (dys)regulation: a systematic review and meta-analysis. Clin Psychol Rev.

[14] Miola A, Cattarinussi G, Antiga G, Caiolo S, Solmi M, Sambataro F (2022). Difficulties in emotion regulation in bipolar disorder: a systematic review and meta-analysis. J Affect Disord.

[15] Lawlor C, Hepworth C, Smallwood J, Carter B, Jolley S (2020). Self-reported emotion regulation difficulties in people with psychosis compared with non-clinical controls: a systematic literature review. Clin Psychol Psychother.

[16] Crowell SE, Beauchaine TP, Linehan MM (2009). A biosocial developmental model of borderline personality: elaborating and extending Linehan’s Theory. Psychol Bull.

[17] Soler J, Elices M, Pascual JC, Martin-Blanco A, Feliu-Soler A, Carmona C (2016). Effects of mindfulness training on different components of impulsivity in borderline personality disorder: results from a pilot randomized study. Borderline Personal Disord Emot Dysregulation..

[18] Carmona iFarres C, Elices M, Soler J, Dominguez-Clave E, Pomarol-Clotet E, Salvador R (2019). Effects of mindfulness training on borderline personality disorder: impulsivity versus emotional dysregulation. Mindfulness.

[19] Iliakis EA, Sonley AK, Ilagan GS, Choi-Kain LW (2019). Treatment of borderline personality disorder: is supply adequate to meet public health needs?. Psychiatr Serv.

[20] Stepp SD, Epler AJ, Jahng S, Trull TJ (2008). The effect of dialectical behavior therapy skills use on borderline personality disorder features. J Personal Disord.

[21] Lindenboim N, Comtois KA, Linehan MM (2007). Skills practice in dialectical behavior therapy for suicidal women meeting criteria for borderline personality disorder. Cogn Behav Pract.

[22] Crane RS, Brewer J, Feldman C, Kabat-Zinn J, Santorelli S, Williams JMG (2017). What defines mindfulness-based programs? The warp and the weft. Psychol Med.

[23] Eeles J, Walker DM (2022). Mindfulness as taught in dialectical behaviour therapy: A scoping review. Clin Psychol Psychother.

[24] Farb NAS, Anderson AK, Irving JA, Segal ZV (2014). Mindfulness interventions and emotion regulation. Handbook of emotion regulation.

[25] Lewis JJ (2018). Attachment insecurity, emotion regulation difficulties, and mindfulness deficits in personality pathology.

[26] Rivera AC (2014). Mindfulness and self-compassion in relation to borderline personality disorder.

[27] Wupperman P (2007). Are deficits in mindfulness core features of borderline personality disorder?.

[28] Selby EA, Fehling KB, Panza EA, Kranzler A (2016). Rumination, mindfulness, and borderline personality disorder symptoms. Mindfulness.

[29] O’Toole SK, Diddy E, Kent M (2012). Mindfulness and Emotional Well-being in Women with Borderline Personality Disorder. Mindfulness.

[30] Didonna F, Rossi R, Ferrari C, Iani L, Pedrini L, Rossi N (2019). Relations of mindfulness facets with psychological symptoms among individuals with a diagnosis of obsessive-compulsive disorder, major depressive disorder, or borderline personality disorder. Psychol Psychother-Theory Res Pract.

[31] Perroud N, Nicastro R, Jermann F, Huguelet P (2012). Mindfulness skills in borderline personality disorder patients during dialectical behavior therapy: preliminary results. Int J Psychiatry Clin Pract.

[32] Mitchell R, Roberts R, Bartsch D, Sullivan T (2019). Changes in mindfulness facets in a dialectical behaviour therapy skills training group program for borderline personality disorder. J Clin Psychol.

[33] Krantz LH, McMain S, Kuo JR (2018). The unique contribution of acceptance without judgment in predicting nonsuicidal self-injury after 20-weeks of dialectical behaviour therapy group skills training. Behav Res Ther.

[34] Hölzel BK, Lazar SW, Gard T, Schuman-Olivier Z, Vago DR, Ott U (2011). How does mindfulness meditation work? Proposing mechanisms of action from a conceptual and neural perspective. Perspect Psychol Sci.

[35] Kounidas G, Kastora S (2021). Mindfulness training for borderline personality disorder: a systematic review of contemporary literature. Personal Ment Health.

[36] Hepgul N, King S, Amarasinghe M, Breen G, Grant N, Grey N (2016). Clinical characteristics of patients assessed within an Improving Access to Psychological Therapies (IAPT) service: Results from a naturalistic cohort study (Predicting Outcome Following Psychological Therapy; PROMPT). BMC Psychiatry.

[37] NHS workforce statistics. NHS Digital. Available from: https://digital.nhs.uk/data-and-information/publications/statistical/nhs-workforce-statistics. [Cited 2022 May 20].

[38] National Collaborating Centre for Mental Health (Great Britain) Staff, National Library of Medicine (2009). Borderline Personality Disorder: the NICE Guideline on Treatment and Management.

[39] Bohus M, Kleindienst N, Limberger MF, Stieglitz RD, Domsalla M, Chapman AL (2009). The short version of the Borderline Symptom List (BSL-23): development and initial data on psychometric properties. Psychopathology.

[40] Kleindienst N, Jungkunz M, Bohus M (2020). A proposed severity classification of borderline symptoms using the borderline symptom list (BSL-23). Borderline Personal Disord Emot Dysregulation.

[41] Preece DA, Becerra R, Robinson K, Dandy J, Allan A (2018). Measuring emotion regulation ability across negative and positive emotions: The Perth Emotion Regulation Competency Inventory (PERCI). Personal Individ Differ.

[42] Preece DA, Becerra R, Sauer-Zavala S, Boyes M, McEvoy P, Villanueva C (2021). Assessing emotion regulation ability for negative and positive emotions: Psychometrics of the Perth Emotion Regulation Competency Inventory in United States Adults. J Affect Disord.

[43] Baer R (2019). Assessment of mindfulness by self-report. Curr Opin Psychol.

[44] Baer RA, Smith GT, Hopkins J, Krietemeyer J, Toney L (2006). Using self-report assessment methods to explore facets of mindfulness. Assessment.

[45] Gu J, Strauss C, Crane C, Barnhofer T, Karl A, Cavanagh K, Kuyken W (2016). Examining the factor structure of the 39-item and 15-item versions of the Five Facet Mindfulness Questionnaire before and after mindfulness-based cognitive therapy for people with recurrent depression. Psychol Assess..

[46] Caletti E, Pagliari C, Vai B, Delvecchio G, Brambilla P (2020). Which are the best questionnaires to longitudinally evaluate mindfulness skills in personality disorders?. J Affect Disord.

[47] Hayes AF. Introduction to Mediation, Moderation, and Conditional Process Analysis: Third Edition: A Regression-Based Approach. New York: Guilford Press; 2022.

[48] Fritz MS, MacKinnon DP (2007). Required sample size to detect the mediated effect. Psychol Sci.

[49] Field A. Discovering statistics using IBM SPSS statistics. London: Sage; 2013.

[50] Diedenhofen B, Musch J (2015). cocor: a comprehensive solution for the statistical comparison of correlations. PLoS One.

[51] van der Kaap-Deeder J, Brenning K, Neyrinck B (2021). Emotion regulation and borderline personality features: the mediating role of basic psychological need frustration. Personal Individ Differ.

[52] Martino F, Caselli G, Di Tommaso J, Sassaroli S, Spada MM (2018). Anger and depressive ruminations as predictors of dysregulated behaviours in borderline personality disorder. Clin Psychol Psychother.

[53] Graling K. The impact of emotion regulation and interpersonal problems on behavioral dysregulation in a college student sample: An investigation of the mediating role of mentalizing. Vol. 74, Issue 9-B(E), Dissertation Abstracts International: Section B: The Sciences and Engineering.

[54] Schramm AT, Venta A, Sharp C (2013). The role of experiential avoidance in the association between borderline features and emotion regulation in adolescents. Personal Disord Theory Res Treat.

[55] Schmidt C, Soler J, Farrés CCI, Elices M, Domínguez-Clavé E, Vega D (2021). Mindfulness in borderline personality disorder: Decentering mediates the effectiveness. Psicothema.

[56] Kazdin AE (2009). Understanding how and why psychotherapy leads to change. Psychother Res.

[57] Bolton W, Lovell K, Morgan L, Wood H, Barrett J, Castillo H, et al. Meeting the Challenge, Making a Difference. London: Department of Health; 2014 p. 84.

